# Functional and Radiological Outcomes after Treatment with Custom-Made Glenoid Components in Revision Reverse Shoulder Arthroplasty

**DOI:** 10.3390/jcm11030551

**Published:** 2022-01-22

**Authors:** Reinhold Ortmaier, Guido Wierer, Michael Stephan Gruber

**Affiliations:** 1 Department of Orthopedic Surgery, Ordensklinikum Linz Barmherzige Schwestern, Vinzenzgruppe Center of Orthopedic Excellence, Teaching Hospital of the Paracelsus Medical University Salzburg, 4020 Linz, Austria; r.ortmaier@gmail.com; 2 Department of Orthopedics and Traumatology, Paracelsus Medical University Salzburg, 5020 Salzburg, Austria; wierer@gmail.com; 3 Research Unit for Orthopaedic Sports Medicine and Injury Prevention, Institute for Sports Medicine, Alpine Medicine and Health Tourism, UMIT, 6060 Hall in Tirol, Austria; 4 Trauma Center Linz, Teaching Hospital of the Paracelsus Medical University Salzburg, 5020 Salzburg, Austria

**Keywords:** revision total shoulder arthroplasty, reverse total shoulder arthroplasty, glenoid bone loss, custom-made prosthesis

## Abstract

Glenoid implant position and fixation are challenging in severe glenoid defects in reverse total shoulder arthroplasty (rTSA). Custom-made glenoid implants are metal augmented implants that are specially produced for a certain defect. They provide the restoration of the joint line and proper fixation. This retrospective data analysis investigated the clinical and radiological outcomes after revision using custom-made glenoid implants. Between 2018 and 2020, nine patients (10 shoulders) with severe glenoid defects underwent revision rTSA using a custom-made glenoid implant (Materialise Glenius or Lima ProMade). The pre- and postoperative Constant Murley Score (CMS), UCLA Score and Subjective Shoulder Value (SSV) were assessed. Postoperative CT scans and X-rays in two planes were available. The minimum follow-up was 12 months, with a mean follow-up of 23.1 months. The mean preoperative CMS, UCLA Score and SSV were 10.9, 4.1 and 11.0, respectively. The mean postoperative CMS, UCLA Score and SSV showed significant increases of 51.7 (<0.001), 22.9 (<0.001) and 52.0 (<0.001), respectively. There were no signs of loosening implants or scapular notching, and no revision was necessary. This trial showed promising clinical and radiological short-term outcomes for custom-made glenoid components in revision rTSA.

## 1. Introduction

Reverse total shoulder arthroplasty (rTSA) has proven very successful, and an increase in annual surgery is expected in the coming years [1]. However, revision surgery will be needed more often alongside the more frequent use of rTSA. Despite good functional results, complications occur in up to 15% of cases when used in the primary setting and up to 40% of cases when used in the revision setting [2].

One of the most challenging complications surgeons face with rTSA is severe glenoid defects. Glenoid defects classically occur after the infection or loosening of the glenoid implant after total shoulder arthroplasty (TSA) due to cuff arthropathy or rheumatoid arthritis [3].

When implanting a glenoid component in rTSA, the major goals are to fix the glenoid component properly and with adequate orientation. Orientation comprises version, inclination and mediolateral expansion, together defining the joint line.

Glenoid bone defects can be described using several classifications [4,5]. Summarizing the different classifications presented in the literature, one can divide the defects depending on the state of the glenoid rim and vault into contained, non-contained and complex defects.

Strategies for the treatment and reconstruction of glenoid bone defects include reaming and/or bone grafting using auto or allografts and metal augments, respectively [5,6]. Custom-made implants can be used in cases of severe, complex glenoid defects. They come with a patient-specific shape that fits into the defect and represents an interesting treatment option for those patients [7]. The missing scapular bone and the original joint line are reconstructed with the aid of statistical shape modelling [8]. The main benefit of custom-made prostheses is their defect-specific shape and hence suitability for extensive bone loss. Other considerable benefits comprise exact planning for screw direction and length and correct positioning due to implant guides. On the downside, custom-made implants are more expensive than conventional implants, and the greater distance between the center of rotation and the bone/metal-line may cause more shearing forces and more implant loosening.

This study was designed to evaluate the clinical and radiological outcomes of patients who underwent revision-rTSA using custom-made glenoid implants for severe glenoid defects.

## 2. Materials and Methods

This retrospective data analysis included nine patients (10 shoulders) who were treated with revision rTSA using a custom-made glenoid implant to address the bone defect by a single senior surgeon in a single institution between 2018 and 2020. All patients were women, with a mean age of 76.6 years at the time of surgery (range, 65 to 83).

The mean follow-up was 23.1 months after surgery (range, 16 to 30), with a minimum follow-up of 12 months. Table 1 show patient demographics in detail.

Defects of the glenoid were classified according to Kocsis et al. [5]. The evaluations were based on preoperative X-rays in the anteroposterior and axial directions or on a CT scan and divided the defects into three types. Important landmarks for classification are the lateral base of the coracoid and the medial spinoglenoid notch. Type 1 describes a defect with the most medial point of the intact glenoid being at least at the level of the base of the coracoid. In a Type 2 defect, the most medial point of the intact glenoid lays between the base of the coracoid and the medial spinoglenoid notch. A Type 3 defect describes a case in which the most medial point of the intact glenoid is at or medial to the spinoglenoid notch. In this study, there were four patients with Type 2 defects and six patients with Type 3 defects.

Two different types of implants were used for all patients. The glenoid implant designed by Materialise (Glenius, Materialise NV, Leuven, Belgium) was used in eight cases in combination with the humeral component of the Delta XTEND system (Depuy Synthes, Warsaw, Poland). Two patients received a glenoid implant by Lima (ProMade; LimaCorporate, Udine, Italy), together with Lima’s humeral component. The implants consist of a metal base plate and, depending on the defect size, with a supporting, porous structure filling the bone defect (titanium alloy). The glenosphere connector is integrated into the baseplate. The manufacturing and delivery time from the point of the surgeon’s approval is approximately six weeks. The costs vary from 10,000€ to 15,000€ per package, consisting of planning, original implant, scapula and implant trials and drill and positioning guides.

The reason for surgery using a custom-made glenoid implant was pre-existing surgeries in all cases and hence, bone loss. The patients had a mean of 1.3 (range, 1 to 2) surgeries prior to the index surgery.

Clinical evaluation was conducted before and after surgery using the CMS (Constant Murley Score), SST (Simple Shoulder Test) and UCLA (University of California at Los Angeles) Shoulder Score.

Radiological examinations consisted of X-rays in two planes (AP and axial or Y-view) before and after surgery as well as CT scans preoperatively and, if the patients consented, at the final follow-up. A postoperative CT scan was used to evaluate the ingrowth of the implant and to assess the matching of the planned position with the actual position of the glenoid implant. Furthermore, a comparison between the planned and actual orientation and length of the screws was performed in these cases.

**Figure 1:**
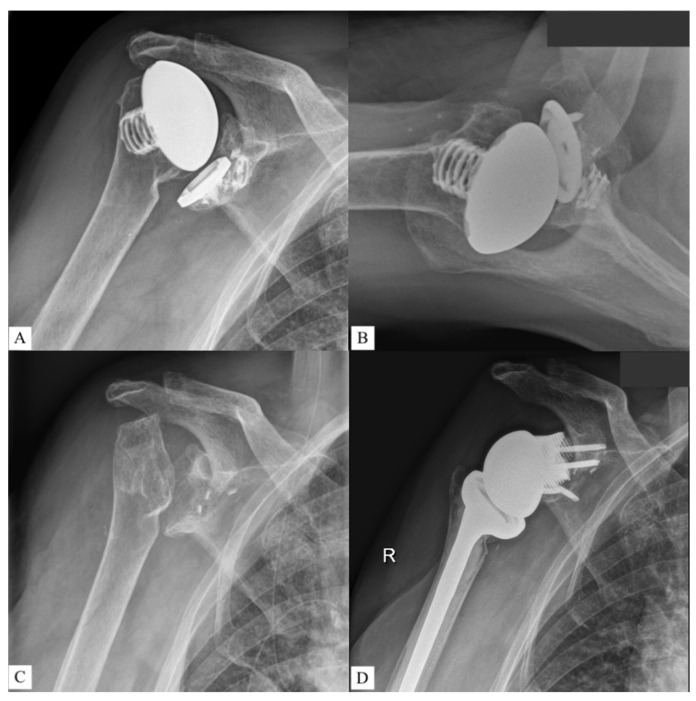
One case of revision reverse total shoulder arthroplasty severely damaged glenoid in the setting of implant loosening after anatomical TSA. Preoperative X-rays were performed in the (**A**) anteroposterior and (**B**) axial directions. (**C**) The surgical procedure consisted of two-time revision with explantation of the anatomical prosthesis and exclusion of infection via sonication and tissue samples. (**D**) Surgery using a custom-made glenoid component was performed after confirmation of aseptic loosening.

**Figure 2:**
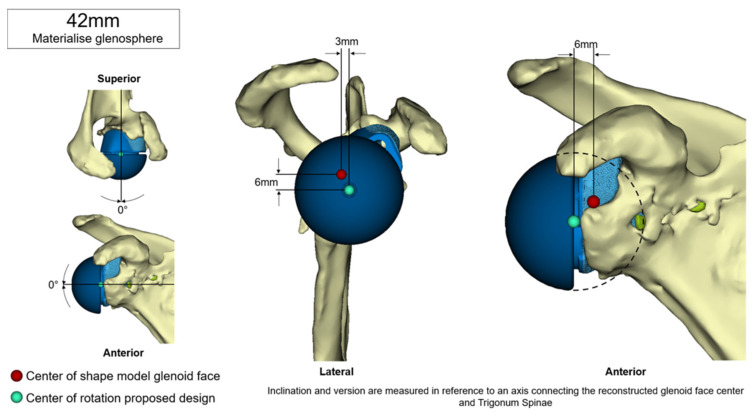
Restoration of the original glenoid surface via statistical shape modelling. Source: planning report provided by Materialise NV, Leuven, Belgium. Reprinted with permission from Materialise. ©2021 Materialise NV.

**Figure 3:**
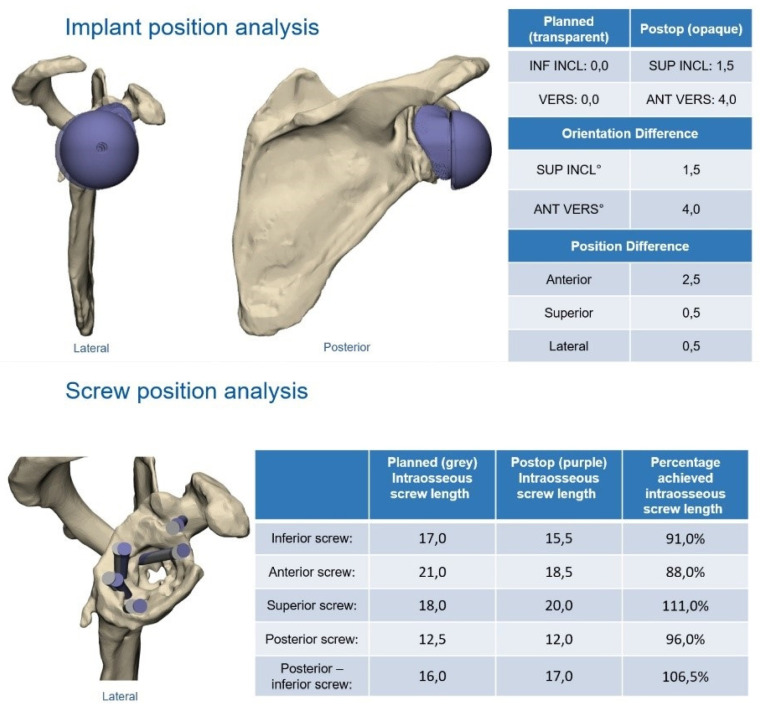
The postoperative report shows little difference between the planned and actual positions. Source: planning report provided by Materialise NV, Leuven, Belgium. Reprinted with permission from Materialise. ©2021 Materialise NV.

Figure 1, Figure 2 and Figure 3 show an example procedure on a case with severe bone loss due to glenoid loosening after primary anatomical TSA. Septic loosening must be excluded using a two-time procedure with sonication and tissue sampling. Radiographs in the anteroposterior and axial directions and an additional CT scan are necessary before surgery. The CT scan must be performed according to the manufacturer’s specifications to assess the bone stock and plan an adequate prosthesis. The original glenoid is reconstructed using statistical shape modelling, and an appropriate centre of rotation is proposed [8]. The position of the screws is proposed depending on the best direction and maximum possible length of the intraosseous screw length according to bone stock and quality.

Statistical analyses were performed with IBM SPSS Statistics (Windows, 64-bit, version 23.0; IBM Corp., Armonk, NY, USA). Patient characteristics were examined using descriptive statistics. Paired *t*-tests were used to compare pre- and postoperative clinical scores. Paired *t*-tests and descriptive statistics were used to evaluate the radiological findings. Statistical significance was set at *p* ≤ 0.05.

This retrospective data analysis was approved by the Ethics Committee of the medical university of JKU, Linz.

## 3. Results

All clinical scores improved significantly from pre- to postoperatively: CMS from 10.9 to 51.7 points (*t* = −13.688, *p* < 0.001), UCLA Score from 4.1 to 22.9 points (*t* = −15.204, *p* < 0.001) and SSV from 11 to 52 percent (*t* = −14.298, *p* < 0.001). The details of the clinical evaluation are shown in Table 2 and Table 3.

Six patients consented to a follow-up CT scan after the surgery. The mean differences between the planned and the actual inclination and retroversion of the glenoid implant were 2.1 degrees (range, 1 to 3) and 3.4 degrees (range, 2 to 7.5), respectively. The posterior alignment showed a mean difference of 2.9 mm (range, 2 to 4), the superior alignment was at a mean of 0.9 mm different from the planned position (range, 0.5 to 1.5) and the mean difference of the medial alignment was 0.5 mm (range, 0.5 to 0.5). Table 4 show the details of the differences in the implant position.

Radiological follow-up enabled an evaluation of the planned screw positions and length (Table 5). There was no difference in the orientation of the planned screws compared to the screws used. The measurement of the intraosseous length of the screws showed a mean accuracy of 95.4% (range, 77.3 to 107.5). There were no signs of loosening implants or scapular notching, and no revision was necessary.

**Figure 4:**
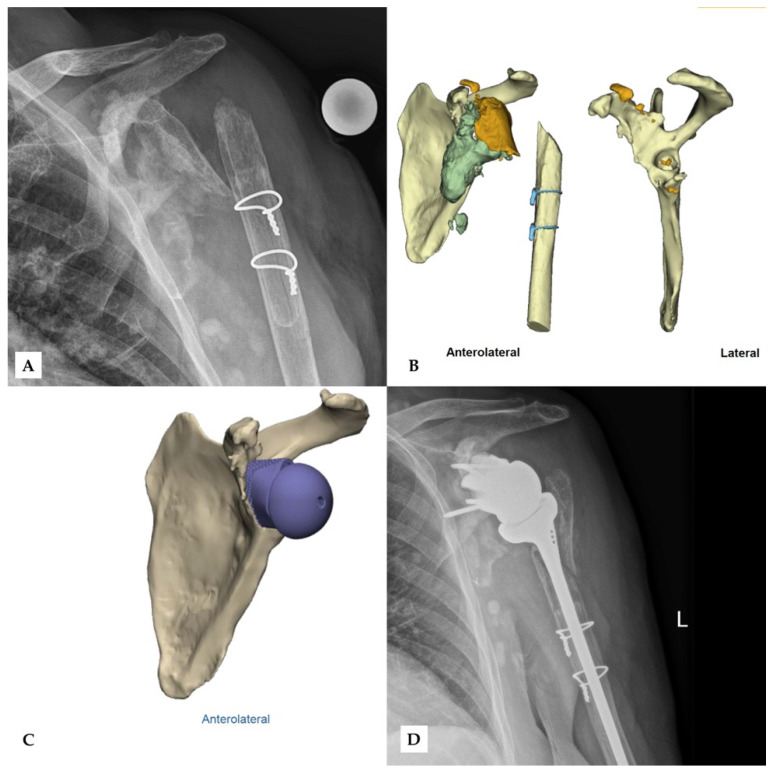
Second case of revision rTSA. The preoperative X-ray (**A**) after removal of the prior implant shows extensive damage of the glenoid and the proximal humerus. Bone fragments (orange) and excess cement (grey) can be seen in the CT-scan (**B**), while the preoperative model (**C**) shows the scapula after the removal of these fragments. The final outcome shows the glenoid implant in the correct position (**D**). Reprinted with permission from Materialise. ©2021 Materialise NV.

**Figure 5:**
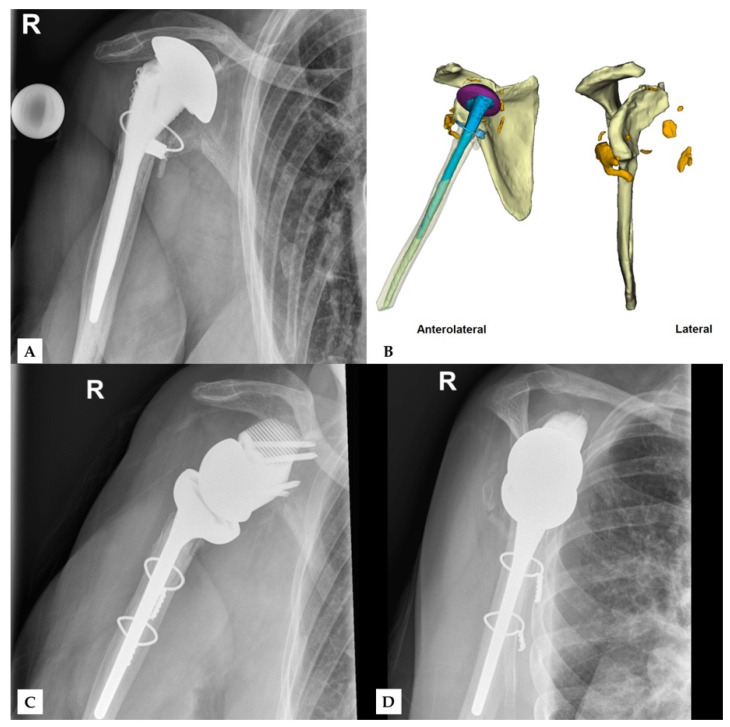
Third case of a revision rTSA. The preoperative situation is displayed in the images on the top (**A**,**B**). The bottom images show the outcome in AP (**C**) and lateral direction (**D**). Reprinted with permission from Materialise. ©2021 Materialise NV.

Finally, no complications were observed during surgery or in follow-up. Figure 4 and Figure 5 show two patients who received revision rTSA. The patients are very happy with the outcome. Considering the devastating initial situation, the comparison between preoperative X-rays and postoperative X-rays shows an excellent result.

## 4. Discussion

Different options for revision arthroplasty of the shoulder joint are of great interest as a consequence of the increasing prevalence of total shoulder arthroplasty. Hence, the present study aims to evaluate the outcome of novel custom-made glenoid components in revision rTSA. The assessment of custom-made implants showed significant results regarding clinical and radiological outcomes.

One of the first trials investigating custom rTSA was conducted by Chammaa et al. in 2017 [13]. They showed the reliability and a significant effect of custom-made implants on 37 patients. The mean Oxford shoulder score showed a significant increase from 11 to 27 points, and the SSV increased from 23% to 60%. However, they also faced complications in 24% of the cases. In general, the clinical improvement does match our results, but unlike the current study, they examined the influence of the implant only in the setting of primary shoulder arthroplasty. Porcellini et al. performed follow-up on six patients who underwent rTSA using custom-made glenoid components due to severe combined defects [7]. They observed increases in all clinical parameters (CMS improved by 9.83 points, ASES score by 30.57 points), but no information regarding statistical significance was provided. Other studies by Bodendorfer et al. (11 patients, significant improvements in range of motion, no complications) and DeBeer et al. (10 patients, postoperative CMS 41.3 points, complications in 20%) came to the same conclusion [3,14]. DeBeer et al. also performed a radiological evaluation, which investigated the mean difference between planned and postoperative implant version and inclination (6° ± 4° and 4° ± 4°, respectively) [3]. The three described studies have in common that they were conducted as multicentre studies. One single-centre trial performed by Rangarajan et al. in 2020 examined the novel system in 18 patients who underwent primary and revision surgery [15]. They showed significant improvements in all clinical scores, such as the Constant Score (24.6 points preoperatively, 60.4 points postoperatively) and in mean forwards flexion and abduction, but not external rotation. The radiological investigation showed no evidence for implant loosening or other hardware failure. Despite good clinical and radiological outcomes, they observed complications in 21% of the patients.

The current clinical assessment showed significant increases in CMS, UCLA score and SSV at a mean follow-up of 23.1 months. The radiological assessment proved a mean accuracy of 93.4% with the comparison of planned to used screw length. The average deviations from the planned implant position to the actual position were 2.9 mm in the posterior, 0.9 mm in the superior and 0.5 mm in the medial directions. In addition, no loosening or fracture was observed. These clinical and radiological results match the existing literature as described. However, the complication rate was lower in this trial.

The currently available custom-made glenoid components are compatible with an anatomical shoulder prosthesis and a reverse total shoulder prosthesis [16]. One challenge in rTSA is the distance between the COR and the bone/metal-line of the scapula. The greater the distance, the greater the shearing forces and thus loosening of the glenoid component. As a result of the attempt to restore the original joint line, one experiences an increase in the distance of COR to the bone/metal line. However, we did not observe an increase in glenoid loosening. This may be due to the following three important factors: (1) The mesh material in combination with the coating leads to stable integration. This effect was also seen in a trial investigating custom-made acetabular components in total hip arthroplasty using the same material [17]. (2) The prosthesis is customized to the defect surface, which leads to a maximized area of contact. (3) The positioning of the screws is essential. Not only does the divergent direction lead to good primary stability [16,18], but preoperative planning using a CT scan allows for the assessment of the bone stock and density and thus the selection of the best possible direction. An algorithm used by the planning committee suggests the highest possible number of screws with the proposed direction. The proposition is dependent on a maximum screw length in the densest bone areas and in the diverging direction.

The major strength of this study is that all treatments included rTSA in the setting of revision surgery with a considerable shift between COR and the bone/metal line, and all surgeries were carried out by one surgeon, resulting in a homogeneous study population. We did not observe any loosening in our follow-up, which may be due to the key factors mentioned. Longer follow-up, as well as a greater study population, is necessary to draw better conclusions about glenoid implant loosening. However, due to the novelty of this technique and its’ restricted indication, only a few patients have received custom made glenoid implants so far, and long term follow-up is not yet available.

## 5. Conclusions

Based on our results, custom-made glenoid components present an adequate solution for severe glenoid defects in revision reverse total shoulder arthroplasty. The short-term survival and clinical follow-up data are promising.

However, long-term outcomes concerning the survival rate or economic issues of custom-made glenoid components have not been reported. Nevertheless, these findings represent an important and, often, the only reasonable option in revision settings with massive bone loss.

## Figures and Tables

**Table 1:** Patient demographics (n = 10) ^a^.

Sex	Side	Age (y)	Follow-Up (m)	ASA	Bone Defect ^b^
f: 10 (100) m: 0 (0)	Left: 3 (30) Right: 7 (70)	Mean 76.6 SD ±5.6	Mean 23.1 SD ±4.7	ASA 1: 0 (0) ASA 2: 6 (60) ASA 3: 4 (40) ASA 4: 0 (0) ASA 5: 0 (0) ASA 6: 0 (0)	Type 1: 0 (0) Type 2: 4 (40) Type 3: 6 (60)

^a^ Data are presented as absolute numbers [percentages] unless otherwise indicated. y, years; m, months; SD, standard deviation; ASA, American Society of Anaesthesiologists [9]. ^b^ Bone defect scoring according to Kocsis [5].

**Table 2:** Clinical Scores ^a^.

	CMS ^b^			UCLA ^c^			SSV ^d^		
Patient	preOP	postOP	*p*-Value ^e^	preOP	postOP	*p*-Value ^e^	preOP	postOP	*p*-Value ^e^
1	14	64		4	22		10	60	
2	11	69		2	31		15	70	
3	16	52		7	21		10	50	
4	17	49		5	24		15	50	
5	15	50		5	23		10	60	
6	6	41		5	22		10	50	
7	8	37		2	20		5	30	
8	8	59		4	24		5	50	
9	8	47		3	20		15	50	
10	6	49		4	22		15	50	
Mean	10.9	51.7	<0.001	4.1	22.9	<0.001	11.0	52.0	<0.001
± SD	±4.25	±9.86		±1.52	±3.18		±3.94	±10.33	

^a^ Data are presented as score values unless otherwise indicated. SD, Standard Deviation. ^b^ CMS, Constant Murley Score: 0–100 points [10]. ^c^ UCLA, University of California at Los Angeles: 0–35 points [11]. ^d^ SSV, Subjective Shoulder Value: 0–100% [12]. ^e^ Paired *t*-test.

**Table 3:** Constant Murley Score detailed values ^a^.

	Abduction, deg			Anteversion, deg			Strength, kg ^b^		
Patient	preOP	postOP	*p*-Value ^c^	preOP	postOP	*p*-Value ^c^	preOP	postOP	*p*-Value ^c^
1	30	170		30	170		0	2	
2	20	160		40	170		0	2	
3	10	110		20	120		0	2	
4	20	120		20	120		0	3	
5	30	130		40	140		0	1	
6	20	140		30	150		0	3	
7	10	60		30	90		0	3	
8	20	110		40	140		0	1	
9	10	100		10	100		0	3	
10	20	110		20	120		0	2	
Mean	19	121	<0.001	28	132	<0.001	0	2.2	<0.001
± SD	±7.38	±31.43		±10.33	±27		±0	±0.79	

^a^ Data are presented as degrees and kilograms. SD, Standard Deviation; CMS, Constant Murley Score; UCLA, University of California at Los Angeles; SSV, Subjective Shoulder Value. ^b^ Maximum strength using a tensiometer, upper limb in 90° abduction, palm facing downwards. ^c^ Paired *t*-test.

**Table 4:** Differences in implant position compared to the planning report ^a^.

	Difference of Orientation (deg)		Difference of Position (mm)		
Patient	Inferior Inclination	Retroversion	Posterior	Superior	Medial
2	2	7.5	4	0.5	0.5
3	3	2	3	1.5	0.5
4	2	3	2	1	0.5
5	1	2	3	0.5	0.5
7	3	2	3	1.5	0.5
8	1.5	4	2.5	0.5	0.5
Mean	2.1	3.4	2.9	0.9	0.5
±SD	±0.8	±2.2	±0.7	±0.5	±0.0

^a^ All measurements are rounded to the nearest 0.5 mm and 0.5%. SD, Standard Deviation.

**Table 5:** Comparison of planned to used screw lengths ^a^.

Patient	Nr. of Screws	Planned Screw Length (mm)	Used Screw Length (mm)	% ^b^
2	5	25.5, 21, 26, 23.5, 15	25.5, 18, 28.5, 21, 14.5	98.3
3	5	27, 14, 20, 31.5, 16.5	29.5, 13.5, 3, 35, 9	77.3
4	5	24, 15, 22, 30, 15	26, 15, 24, 30, 18	107.5
5	5	22, 18, 24, 32, 18	20, 18, 24, 34, 20	101.5
7	5	24, 12, 18, 28, 12	20, 10, 14, 30, 10	89.4
8	5	17, 21, 18, 12.5, 16	15.5; 18.5; 20, 12, 17	98.5

^a^ All measurements are rounded to the nearest 0.5 mm and 0.5%. Measurement of the length of the screws along the core. ^b^ Measurement of the percentage of the planned compared to the actual overall intraosseous screw length.

## Data Availability

The data presented in this study are available on request from the corresponding author. The data are not publicly available due to ethical restrictions.
